# False negative effect of high triglycerides concentration on vitamin D levels: A big data study

**DOI:** 10.5937/jomb0-40106

**Published:** 2023-03-15

**Authors:** Murat Çağlayan, Ataman Gonel, Tugba Songul Tat, Osman Celik, Fidanci Ali Aykut, Ayvali Mustafa Okan, Ulgu Mustafa Mahir, Naim Ata, Suayip Birinci

**Affiliations:** 1 Health Science University, Diskapi Yildirim Beyazit Training and Research Hospital, Department of Medical Biochemistry, Ankara, Turkey; 2 Hasan Kalyoncu University, Faculty of Health Science, Department of Nutrition and Dietetics, Sahinbey, Gaziantep, Turkey; 3 Hasan Kalyoncu University, Gaziantep Medical Point Hospital, Vocational School, First and Emergency Aid Program, Allergy and Immunology Unit, Şehitkamil/Gaziantep, Turkey; 4 Republic of Turkey Ministry of Health, Ankara, Turkey; 5 Üniversiteler Quarter., Bilkent campus, Cankaya / Ankara, Turkey

**Keywords:** 25-OH vitamin D, big data, vitamin D deficiency, triglycerides, cholesterol, 25-OH vitamin D, veliki podaci, deficijencija vitamana D, trigliceridi, holesterol

## Abstract

**Background:**

Inaccurate test results may be a reason why vitamin D deficiency is seen as a common problem worldwide. Interferences from the sample matrix during testing are the most important factors in measurement errors. In this study, the relationship between triglycerides and total cholesterol levels and vitamin D levels in Turkey was investigated.

**Methods:**

The 25-hydroxyvitamin D test results and lipid test results studied in Turkey in 2021 were compared. Data were obtained from the Ministry of Health National Health Database. Simultaneously, 25-hydroxyvitamin D, triglyceride, and total cholesterol levels were studied, and 1,135,644 test results were taken as the basis.

**Results:**

In the group of patients with total cholesterol levels between 0-10.33 mmol/L, the proportion of patients below 20 mg/L ranged from 56.8% to 61.8%. In the patient group with cholesterol between 10.36-259 mmol/L, the rate of patients with less than 20 mg/L was between 70.8-100%, while the rate of patients with cholesterol above 100 mg/L was 0%. The mean 25-hydroxyvitamin D level was 20.1 mg/L in the patient group with a total cholesterol level between 0-10.33 mmol/L, and 16 mg/L in the patient group with a cholesterol level above 10.36 mmol/L. The mean 25-hydroxyvitamin D level was 20.11 mg/L in the patient group with triglycerides 0-10.16 mmol/L, and the 25-hydroxyvitamin D level was 12.28 mg/L in the patient group with triglycerides 10.17-113 mmol/L. The proportion of patients with vitamin D levels above 100 mg/L was found to be 0% in the group of patients with triglycerides above 10.17-113 mmol/L.

**Conclusions:**

According to this study, there is a risk of toxicity when administering vitamin D therapy in patients with high cholesterol and triglycerides levels. This study is the first of this size in the literature. High triglycerides and cholesterol levels can cause inaccurate measurement of vitamin D levels, so care should be taken when evaluating these tests.

## Abstract (sr)

**Uvod:**

Netačni rezultati testova mogu biti razlog zašto se nedostatak vitamina D smatra uobičajenim problemom širom sveta. Interferencije iz matrice uzorka tokom testiranja su najvažniji faktori grešaka u merenju. U ovoj studiji istražen je odnos između nivoa triglicerida i ukupnog holesterola i nivoa vitamina D u Turskoj.

**Metode:**

Upoređeni su rezultati testa 25-hidroksivitamina D i rezultati testa lipida koji su proučavani u Turskoj 2021. godine. Podaci su dobijeni iz Nacionalne zdravstvene baze podataka Ministarstva zdravlja. Istovremeno su proučavani nivoi 25-hidroksivitamina D, triglicerida i ukupnog holesterola, a za osnovu su uzeta 1.135.644 rezultata testova.

**Rezultati:**

U grupi pacijenata sa nivoom ukupnog holesterola između 0-10,33 mmol/L, udeo pacijenata ispod 20 mg/L kretao se od 56,8% do 61,8%. U grupi pacijenata sa holesterolom između 10,36-259 mmol/L, stopa pacijenata sa manje od 20 mg/L bila je između 70,8-100%, dok je stopa pacijenata sa holesterolom iznad 100 mg/L bila 0%. Prosečan nivo 25-hidroksivitamina D bio je 20,1 mg/L u grupi pacijenata sa nivoom ukupnog holesterola između 0-10,33 mmol/L i 16 mg/L u grupi pacijenata sa nivoom holesterola iznad 10,36 mmol/L. Prosečan nivo 25-hidroksi vitamina D bio je 20,11 mg/L u grupi pacijenata sa trigliceridima 0-10,16 mmol/L, a nivo 25-hidroksivitamina D je bio 12,28 mg/L u grupi pacijenata sa trigliceridima 10,17-113 mmol. Utvrđeno je da je procenat pacijenata sa nivoom vitamina D iznad 100 mg/L 0% u grupi pacijenata sa trigliceridima iznad 10,17-113 mmol/L.

**Zaključak:**

Prema ovoj studiji, postoji rizik od toksičnosti pri primeni terapije vitaminom D kod pacijenata sa visokim nivoom holesterola i triglicerida. Ova studija je prva ove veličine u literaturi. Visoki nivoi triglicerida i holesterola mogu uzrokovati netačno merenje nivoa vitamina D, tako da treba biti oprezan prilikom procene ovih testova.

## Introduction

Vitamin D deficiency is a very common problem in both developed and developing countries [1] [2] [3] [4]. It is reported that 1 billion people worldwide are exposed to vitamin D deficiency or insufficiency [1]. The fact that vitamin D deficiency is quite common even in regions such as the Middle East, Asia, and South America that are exposed to the sun, and the fact that vitamin D deficiency is seen in many people, suggests that there may be erroneous 25-hydroxyvitamin D (25-OHD) test results [4]. The existence of clinically inconsistent results supports this finding. The fact that some kit manufacturers have changed their vitamin D kits for several generations may be due to their inability to optimize the kits sufficiently. Matrix-induced interferences in the sample during measurement are the most important factors in measurement error [5]. Lipemia has been found to cause erroneous laboratory measurements and to cause negative interference with vitamin D [5] [6] [7] [8]. This negative interference caused by lipemia may hide intoxication levels in patients receiving vitamin D replacement therapy [6]. The aim of this study was to investigate the relationship between triglyceride and total cholesterol levels and vitamin D levels in Turkey.

## Materials and methods

The results of the 25-OHD tests studied in laboratories in Turkey in 2021 were compared with the results of simultaneous blood lipids. Laboratory test results were obtained from the Turkish Ministry of Health National Health Database (THND). To encourage data sharing with the scientific community and scientific research, laboratory results were presented to the author group under the supervision of the Ministry of Health, and various publications were made [9] [10]. The National Health Database includes laboratory service information and test process information in terms of laboratory tests. Test process information consists of the test name, test result, test unit, and reference range. Laboratory service information includes the demographic information of individuals. In the 25-OHD tests studied in Turkey, chromatographic methods were generally used, especially the immunoassay method (approximately 95%). Triglyceride and total cholesterol tests were obtained using various biochemistry auto analysers, and the test results were transferred to the database after they were approved in the laboratory. In this study, synchronized 25-OHD, triglycerides, and total cholesterol levels were studied, and 1,135,644 laboratory test results were taken as the basis in 2021. Samples from each application had the same run number, and the samples were considered to have the same serum properties.

This study was conducted under the Declaration of Helsinki and received approval from the Turkish Ministry of Health with the waiver of informed consent for retrospective data analysis (95741342-020/27112019). The analyses were completed by transferring the study data to the IBM SPSS Statistics (version 26) program. Descriptive statistics (average, standard deviation) were given for the numerical data. Whether there was a difference between the two independent groups was checked with the independent t-test. The results were interpreted by comparing the test statistical results with α=0.05.

## Results

Data from a sample of 1,135,644 patients were included in the study, and patient results performed simultaneously with 25-OHD, triglycerides, and total cholesterol were used. The levels of triglycerides and 25-OHD in the patient samples, and total cholesterol and 25-OHD levels were compared. Total cholesterol levels divided into 10 groups in the range of 0-25.9 mmol/L in 2.59 mmol/L increments, and one group in the range of 25.9-259 mmol/L, for a total of 11 groups. Mean values between 0-10.33 μmol/L (mean±SD) were respectively; 19±13.04 μg/L, 19.51±12.45 μg/L, 20.86±13.37 μg/L, and 20.89±14.22 μg/L, respectively (Table 1). It was observed that 25-OHD levels were lower in groups with total cholesterol levels above 10.36 mmol/L. The 25-OHD level (mean±SD) in the group with total cholesterol levels of 10.36-12.92 mmol/L was 16.76±12.86 μg/L. In the 12.95-15.51 mmol/L group, the 25-OHD level (mean±SD) was 12.89±12.36 μg/L that can be considered as lower. The lowest 25-OHD levels were 8.08±4.54, 3±0 μg/L in the 20.72-23.28 mmol/L and 23.31-25.87 mmol/L groups (Table 1). 25-OHD levels corresponding to total cholesterol levels were 20.1±12.9 μg/L in the range of 0-10.33 mmol/L (mean±SD) and in the range of 10.36-259 mmol/L the levels were 16±12.93 μg/L (mean±SD). The mean 25-OHD level in the range of 10.36-259 mmol/L was found to be quite low (Table 2). In the four groups in the range of 0-10.36 mmol/L, there were 6, 713, 771 and 48 patient samples respectively. There were 1538 patient samples in total, and no intoxication was observed in the range of 10.36-259 mmol/L (Table 2).

**Table 1:** Comparison of Total Cholesterol levels and 25-OHD levels.

Total <br>Cholesterol <br>(mmol/L)	Number of <br>tests	Mean <br>(μg/L)	SD	Number of results with a 25- OHD level below 20 mg/L	Number of results with 25-OHD levels above 100 μg/L	Rate of results with 25-OHD level below 20 μg/L (%)	Rate of results with 25-OHD levels above 100 μg/L (%)
0–2.56	6886	19	13.04	4257	6	61.8	0
2.59–5.15	628757	19.51	12.45	378186	713	60.1	0
5.18–7.74	476261	20.86	13.37	266202	771	55.9	0
7.77–10.33	22687	20.89	14.22	12879	48	56.8	0
10.36–12.92	857	16.76	12.86	607	0	70.8	0
12.95–15.51	124	12.89	12.36	104	0	83.9	0
15.54–18.1	35	13.12	13.07	29	0	82.9	0
18.13–20.69	16	13.72	18.95	14	0	87.5	0
20.72–23.28	7	8.08	4.54	7	0	100	0
23.31–25.87	3	3	0	3	0	100	0
25.9–259	11	12.88	5.94	9	0	81.8	0

**Table 2:** Comparison of Total Cholesterol levels and 25-OHD levels (Values below and above 10.36 mmol/L).

Total <br>Cholesterol <br>(mmol/L)	Number<br>of tests	Mean<br>(μg/L)	SD	Number of results with a 25-OHD level below 20 μg/L	Number of results with 25-OHD levels above 100 μg/L	Rate of results with 25-OHD level below 20 μg/L (%)	Rate of results with 25-OHD levels above 100 μg/L (%)	P<br>value
0–10.33	1134591	20.1	12.9	661524	1538	58.3	0.1	<0.01
10.36–259	1053	16	12.93	773	0	73.4	0	

Triglycerides levels in the range of 0-45.2 mmol/L were divided into 40 groups with 1.12 mmol/L increments, and one group in the range of 45.2-113 mmol/L, for a total of 41 groups. The number of patients with triglycerides levels between 0-1.12 mmol/L was 419424, the number of patients between 1.13-2.25 mmol/L was 510416, and the number of patients between 2.26-3.38 mmol/L was 143196. The number of patients in the other groups varied between 0-40245. The 25-OHD concentrations of the patients varied between 6-20.79 μg/L. In the patient group with the lowest triglycerides level (0-1.12 mmol/L), the lowest rate of patients with less than 20 mg/L was determined as 55.7%. The proportion of patients below 20 μg/L gradually increased up to the triglycerides 12.43-13.55 mmol/L group. Except for one group, the rate of patients below 20 μg/L was found to be between 75% and 100% in all the other groups. The proportion of patients with 25-OHD levels above 100 μg/L was between 0.1%-0.3%, up to the patient group with 9.04-10.16 mmol/L. It was determined to be 0% in the other groups (Table 3).

**Table 3:** Comparison of triglycerides levels with 25-OHD levels.

Triglycerides <br>(mmol/L)	Number<br>of tests	Mean<br>(μg/L)	SD	Number of results with a 25-OHD level below 20 μg/L	Number of results with 25-OHD levels above 100 μg/L	Rate of results with 25-OHD level below 20 μg/L (%)	Rate of results with 25-OHD levels above 100 μg/L (%)
0–1.12	419424	20.79	13.35	233754	649	55.7	0.2
1.13–2.25	510416	20.24	12.91	294744	694	57.7	0.1
2.26–3.38	143196	18.82	11.94	90145	148	63	0.1
3.39–4.51	40245	17.68	11.31	27173	30	67.5	0.1
4.52–5.64	11725	16.99	11.07	8242	10	70.3	0.1
5.65–6.77	4679	15.98	10.08	3475	1	74.3	0.1
6.78–7.9	2259	15.86	10.6	1696	2	75.1	0.1
7.91–9.03	1211	15.25	11.71	947	3	78.2	0.3
9.04–10.16	713	14.8	11.16	576	1	80.8	0.1
10.17–11.29	403	13.6	8.82	332	0	82.4	0
11.3–12.42	283	12.53	8.76	253	0	89.4	0
12.43–13.55	253	11.37	7.14	227	0	89.7	0
13.56-14.68	149	12.12	8.33	218	0	87.6	0
14.69–15.81	140	12.12	8.34	121	0	86.4	0
15.82–16.94	115	12.39	7.97	102	0	88.7	0
16.95–18.07	69	10.86	6.91	61	0	88.4	0
18.08-19.2	53	11.96	7.79	45	0	84.9	0
19.21–20.33	41	12.39	8.43	34	0	82.9	0
20.34–21.46	27	9.81	5.22	26	0	96.3	0
21.47–22.59	21	12.05	12.29	17	0	81	0
22.6–23.72	9	12.25	5.59	8	0	88.9	0
23.73–24.85	10	11.95	4.79	9	0	90	0
25.86–25.98	10	8.66	5.01	10	0	100	0
25.99–27.11	10	15.14	8.66	6	0	60.0	0
27.12–28.24	9	12.41	10.79	8	0	88.9	0
28.25–29.37	10	13.56	6.23	8	0	80	0
29.38–30.5	8	10.55	4.28	8	0	100	0
30.51–31.63	4	8.39	4.35	4	0	100	0
31.64–32.76	6	9.94	4.59	6	0	100	0
32.77–33.89	2	7.77	0.27	2	0	100	0
33.9–35.02	4	9.36	1.28	4	0	100	0
35.03–36.15	4	20.47	8.51	3	0	75	0
36.16–37.28	4	9.37	4.88	4	0	100	0
37.29–38.41	2	6	3	2	0	100	0
38.42–39.54	3	8.69	4.59	3	0	100	0
39.55–40.67	3	7.29	1.96	3	0	100	0
40.68–41.8	NA	NA	NA	NA	NA	NA	NA
41.81–42.93	NA	NA	NA	NA	NA	NA	NA
42.94–44.06	1	18.27	0	1	0	100	0
44.07–45.19	2	14.35	2.35	2	0	100	0
45.2–113	15	6.5	4.38	15	0	100	0

According to Table 4, the number of patients between 0-10.16 mmol/L triglycerides levels was 1,133,868. The number of patients between 10.17-113 mmol/L was determined as 1771. There was a significant difference in vitamin D levels between the two groups (p<0.001). The proportion of patients with vitamin D below 20 μg/L was calculated as 58.3% and 87.1% respectively. The proportion of patients with vitamin D above 100 μg/L was calculated as 0.1% and 0% respectively.

**Table 4:** Comparison of triglycerides levels with 25-OHD levels (Values below and above 10.17 mmol/L).

Triglycerides <br>(mmol/L)	Number<br>of tests	Mean<br>(μg/L)	SD	Number of results with a 25-OHD level below 20 mg/L	Number of results with 25-OHD levels above 100 μg/L	Rate of results with 25-OHD level below 20 μg/L (%)	Rate of results with 25-OHD levels above 100 μg/L (%)	p<br>value
0-10.16	1133868	20.11	12.9	660752	1538	58.3	0.1	<0.001
10.17-113	1771	12.28	8.25	1542	0	87.1	0	

As a result of the Pearson correlation test applied in the group with a triglyceride value less than 10.17 mmol/L, a negative significant relationship was found between 25-OHD and triglyceride (p<0.05). As a result of the Pearson correlation test applied for the group with a triglyceride value of 10.17 mmol/L or more, it was determined that there was no significant relationship between 25-OHD and triglyceride (p>0,05) (Table 5). In the Pearson correlation analysis applied between 25-OHD and total cholesterol; a significant positive correlation was found in those with a total cholesterol value below 10.36 mmol/L; significant negative correlation was found in the group above 10.36 mmol/L (p<0.05) (Table 6).

**Table 5:** Correlation between 25-OHD and Triglycerides. ^*^<0.05

			Triglycerides
Triglycerides<br><10.17 mmol/L	25-OHD	r	-0.073
		p	0.000^*^
		n	1133888
Triglycerides <br>>=10.17 mmol/L	25-OHD	r	0.029
		p	0.216
		n	1776

**Table 6:** Correlation between 25-OHD and total cholesterol. ^*^<0.05

			Total <br>Cholesterol
Total Cholesterol <br><10.36 mmol/L	25-OHD	r	0.059
		p	0.000^*^
		n	1134611
Total Cholesterol <br>>=10.36 mmol/L	25-OHD	r	-0.062
		p	0.011^*^
		n	1053

## Discussion

Vitamin D deficiency has a high prevalence and is associated with musculoskeletal functions as well as many clinical conditions, such as certain malignancies, cardiovascular diseases, infections, obesity, metabolic syndrome, diabetes mellitus, and autoimmune diseases. This has led to a large increase in vitamin D testing worldwide [9] [11]. 25-OHD is the most abundant form of vitamin D in circulation and occurs in the liver. It is used to determine a patient's vitamin D status [1]. There are two types of 25-OHD in circulation: 25-OHD3 (cholecalciferol) which is 95% of 25-OHD, and is of endogenous origin. The other type is 25-OHD2 (ergocalciferol), obtained from plants and fish, which comprises a very small rate in circulation unless vitamin D supplementation is taken [11] [12]. For 25-OHD measurement, immunoassay, HPLC, and liquid chromatography-tandem mass spectrometry (LC-MS/MS) methods were used [13]. Chromatographic methods can effectively distinguish the 25-OHD3 and 25-OHD2 forms that make up 25-OHD, but immunoassay methods are not capable of distinguishing these forms [12] [14]. The most important disadvantage of immunoassays used in vitamin D measurement is that they are affected by many parameters such as endogenous antibodies and xenobiotics in the blood; therefore, and therefore some laboratories prefer to use the LC-MS/MS method [11] [15]. The National Health and Nutritional Examination Survey (NHANES) has recommended LC-MS/MS as the best method for measuring vitamin D metabolites due to its improved sensitivity, accuracy, reproducibility, and high sensitivity [16]. Immunochemical methods are simpler, faster and more cost-effective compared to LC-MS/MS systems [11] [16]. However, with the effect of lipemia and other factors (such as carbohydrates, phospholipids, bile salts, xenobiotics and proteins) that increase the turbidity of the measurement material in the LC-MS/MS technique, ionization may be impaired, and the results may be affected [15]. In immunoassay tests, lipoproteins that block the binding sites on antibodies can affect the antigen-antibody reaction, resulting in false high or false low results [17] [18]. In a case report by Gönel et al. [19], an erroneous low vitamin D result was found in a case in which 25-OHD was measured using the LC-MS/MS method. While the triglycerides level of the patient who was hyperlipidemic was 27.12 mmol/L, vitamin D was measured as 6.4 μg/L and after the dilution study, the level was found to be 188 μg/L. In the same case, it was reported that hypercalcemia and nephrolithiasis developed due to vitamin D replacement at levels higher than needed. When replacement therapy was discontinued, high calcium levels were found to return to normal [19]. In the study by Agarwal et al. [8] on pediatric patients using the immunoassay method, the average of 25-OHD levels to lipemia levels was taken. According to the lipemia levels, the 25-OHD averages are as follows: mild lipemia was 24.7±2.8 μg/L, moderate lipemia was 22.4±3.2 μg/L, and severe lipemia was 20.4±2.7 μg/L, and 25-OHD levels were found to decrease depending on the increase in lipid levels [8]. In a study by Gönel et al. [19] with 100 patients, the 25-OHD3 concentrations of 21 patients were found to be falsely low. The mean 25-OHD level of the initial results of these 21 patients was 9.94 ± 7.85 μg/L. The level after repeated study with dilution was 39.23±18.13 μg/L [15]. Similar results were obtained in our study as well. As total cholesterol and triglyceride levels increased, vitamin D levels were found to be lower. The lowest mean 25-OHD level of the groups with total cholesterol between 0-10.36 mmol/L was 19 μg/L and the mean value was higher. The mean 25-OHD level of the groups with total cholesterol over 10.36 mmol/L was lower, and the highest mean level was found to be 16.76 μg/L (Table 1). It was observed that there was a statistically significant difference between the two groups formed according to values below and above 10.36 mmol/L (p<0.01) (Table 2). In patients with total cholesterol levels below 10.36 mmol/L, the rate of patients with 25-OHD levels below 20 μg/L is 61.8%, while above 10.36 mmol/L, 70.8% and above 20.72 mmol/L, it was 100%. This demonstrates that as the total cholesterol level increases, the rate of vitamin D deficiency increases. In the patient group with a total cholesterol level below 10.36 mmol/L, 1538 patient results showed vitamin D intoxication, while no intoxication was observed above 10.36 mmol/L (Table 2). This indicates that our study supports the case report study of Gönel et al. [19].

The relevance between the effect of lipemia and total cholesterol levels was determined in this study. It has been shown that total cholesterol values above certain levels affect 25-OHD levels. Among the groups formed according to triglycerides levels, the mean levels of 25-OHD were found to be higher in the groups in the 0-10.16 mmol/L range, and lower in the 10.17-113 mmol/L range (Table 3). In the comparison between the two groups, the difference is found to be statistically significant (Table 4) (p<0.001). When comparing triglycerides levels with 25-OHD levels, a total of 41 groups were formed: 40 groups with triglycerides levels between 0-45.19 mmol/L with 1.13 mmol/L increments, and one group between 45.2-113 mmol/L (Table 3). When the 25-OHD mean (mean±SD) of the groups with triglycerides levels between 0-10.16 mmol/L was evaluated, the highest value (mean±SD) in the group with triglycerides levels between 0-1.12 mmol/L was 20.79±13.35 μg/L. Depending on the increase in triglycerides levels, 25-OHD levels decreased regularly. Groups with higher triglycerides levels (>10.17 mmol/L) had lower average 25-OHD levels in general and were found to decrease further with an increase in triglycerides levels (Table 3). Considering the change in 25-OHD levels, the 25-OHD levels of patients with triglycerides levels below and above 10.17 mmol/L were compared (Table 4). The mean of 25-OHD (mean±SD) in patient samples with triglycerides levels in the range of 0-10.16 mmol/L was 20.11±12.9 μg/L, and 12.28±8.25 μg/L in the range of 10.17-113 mmol/L (Table 4). Samples of patients with 25-OHD levels above 100 μg/L (considered vitamin D intoxication) were seen only in groups with a total cholesterol level of 10.33 mmol/L and below. In the range of 0-10.33 mmol/L, there was a total of 1538 patient samples. No intoxication was detected in the range of 10.36-259 mmol/L (Table 3).

The pre-analytical stage is vital to the accuracy of laboratory test results. At the pre-analytical stage, the overall proportion of lipemic samples among all samples, whether inpatient or outpatient, varied between 0.5-2.5% depending on the type of hospital [18]. The most common preanalytical cause of lipemia was the short time between eating and drawing blood. Since it was not possible to adjust this time in patients admitted to the emergency department, the rate of lipemic samples was high. Ambulatory patients need to make appropriate preparations, including fasting, before sampling [18].

It is showed that vitamin D supplementation has a beneficial effect on reducing serum total cholesterol, low density lipoprotein (LDL) cholesterol, and triglycerides levels, but not high density lipoprotein (HDL) cholesterol levels in a review and meta-analys [20]. However according to our study, when vitamin D therapy is to be given, care should be taken in terms of toxicity in patients with high cholesterol and triglycerides.

The subject discussed in this article is to investigate the effect of the presence of cholesterol on the measurement of 25-OHD vitamins. However, it has been suggested that vitamin D may affect endogenous cholesterol synthesis and vice versa. A recovery study is required to say that cholesterol causes this by interacting with 25-OHD at the molecular level or by interfering with the measurement method. According to previously published case report data, it is likelier to say that 25-OHD is affected by high triglycerides as opposed to high cholesterol [19]. The limitations of this study are that the effect of endogenous cholesterol synthesis on the measurement of vitamin D could not be evaluated, and patients who took vitamin D supplements could not be excluded.

## Conclusion

The strength of this study is that it is the first of this size in the literature. Another advantage is that it draws attention to the possibility of intoxication in cases of high cholesterol and triglycerides. Knowing that vitamin D levels may be wrong in the presence of high cholesterol and triglycerides will save time and money. We also conclude from this study that knowing that there may be toxicity, it is necessary to be careful when prescribing vitamin D. A limitation of our study is the absence of dilution measurements.

As a result, high triglycerides and cholesterol levels can cause inaccurate measurement of vitamin D levels, so care should be taken when evaluating these tests.

## Dodatak

### Conflict of interest statement

All the authors declare that they have no conflictof interest in this work.
